# Supplementary figures and images for: Simulation metamodeling approach to complex design of garment assembly lines
Source: PLoS One. 2020 Sep 21;15(9):e0239410. doi: 10.1371/journal.pone.0239410 (PMC7505436; doi:10.1371/journal.pone.0239410)

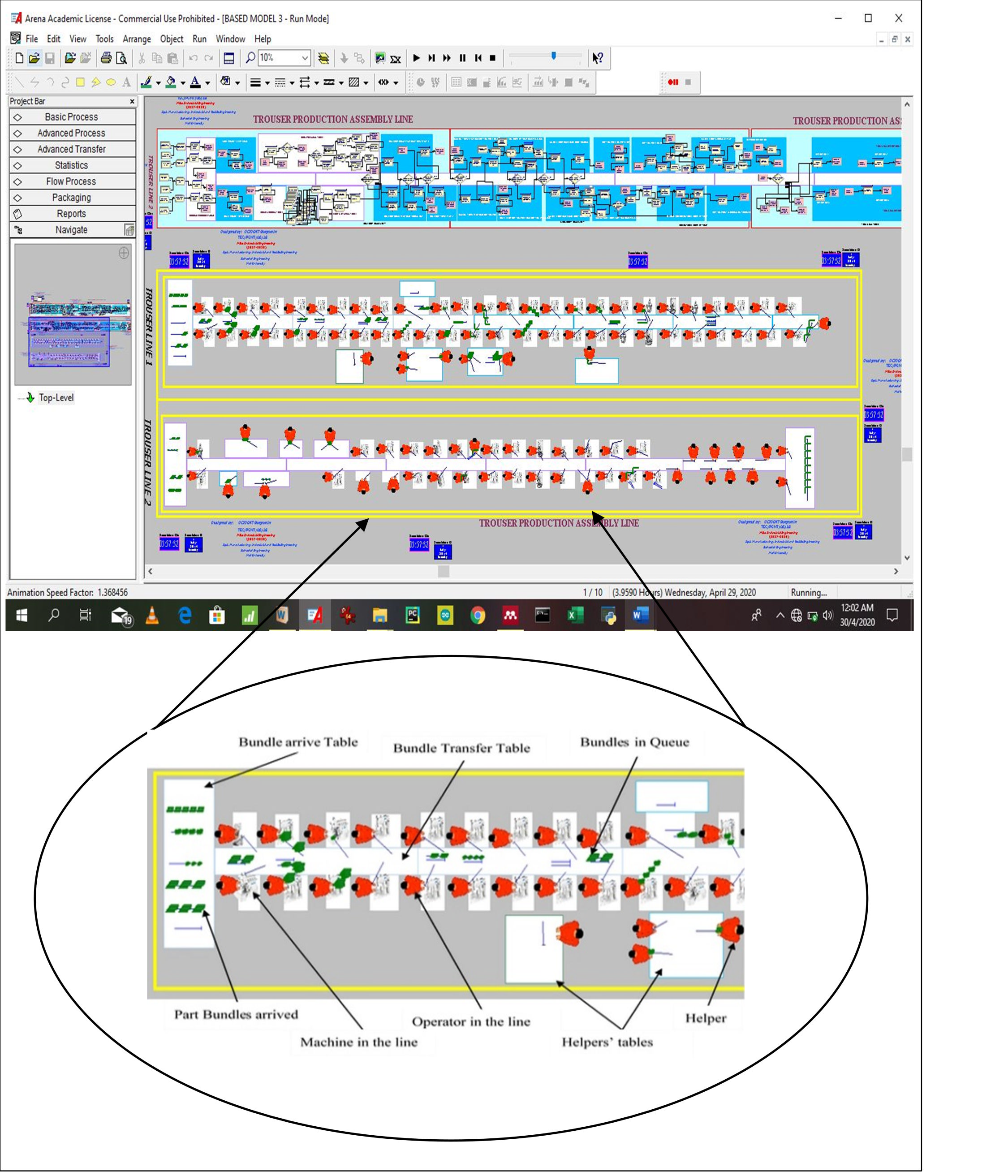

Supplement: S1 Fig — (TIF) [file pone.0239410.s001.tif]

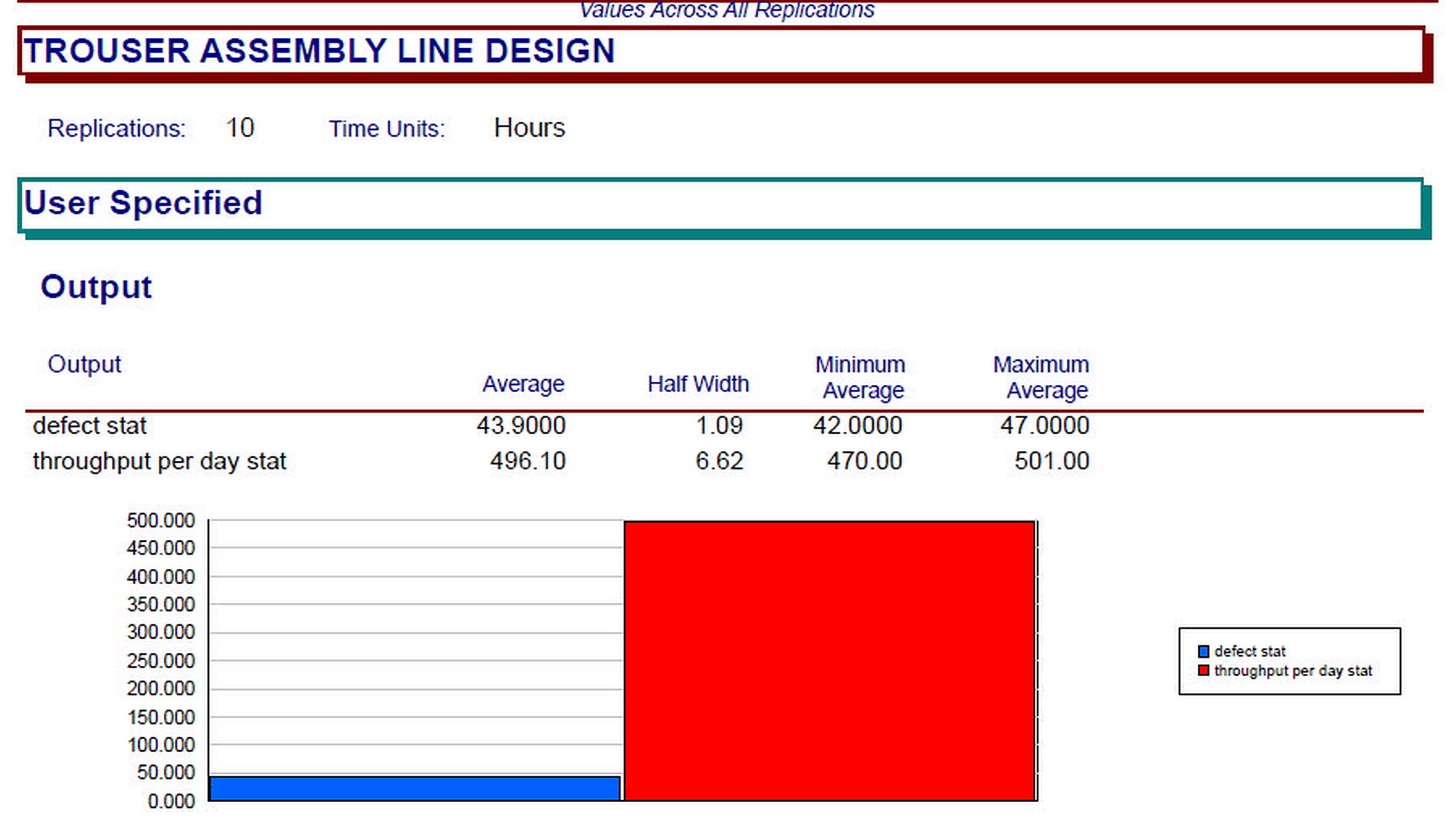

Supplement: S2 Fig — (TIF) [file pone.0239410.s002.tif]
