# Supplementary material for: Simulation metamodeling approach to complex design of garment assembly lines
Source: PLoS One. 2020 Sep 21;15(9):e0239410. doi: 10.1371/journal.pone.0239410 (PMC7505436; doi:10.1371/journal.pone.0239410)
Supplement: S2 Table — (DOCX) [file pone.0239410.s004.docx]

| **OPN^a^** | **Operations description** | **Resource** | **Quantity** | **Processing time distribution per resource** | **Bundle size** |
| --- | --- | --- | --- | --- | --- |
| 1 | Left flybox pressing | Iron press | 1 | TRIA (3, 5.12, 5.9) | 25 |
| 2 | Buttonhole on Left flybox | BH^b^ | 1 | 6.05 + ERLA (0.39, 6) | 25 |
| 3 | Left front rise overlock | O/L^c^ | 1 | 4 + 6.88 * BETA (1.95, 3.37) | 25 |
| 4 | Right front rise overlocks |  |  | 2.29 + ERLA (0.239, 5) | 25 |
| 5 | Knee patch attach | S/NL^d^ | 3 | 20 + 21 * BETA (0.856, 1.33) | 25 |
| 6 | Side pocket flatlock | F/L^e^ | 2 | 4 + 4 * BETA (1.94, 2.74) | 25 |
| 7 | Side pocket overlocks | O/L^c^ | 1 | 2 + ERLA (0.555, 2) | 25 |
| 8 | Right flybox overlock |  |  | 1.6 + LOGN (0.719, 0.418) | 25 |
| 9 | Side pocket attach | S/NL^d^ | 2 | 7 + 11 * BETA (1.67, 1.67) | 25 |
| 10 | Side pocket topstitch | S/NL^d^ | 2 | 10 + GAMM (1.44, 2.7) | 25 |
| 11 | Right flybox attach | S/NL^d^ | 2 | TRIA (13, 20.7, 25) | 25 |
| 12 | Left fly box tacking | S/NL^d^ | 2 | 9 + WEIB (3.39, 2.09) | 25 |
| 13 | Fly attach | S/NL^d^ | 2 | 12.1 + GAMM (0.955, 3.94) | 25 |
| 14 | Front prep bundling | Helper | 1 | 5 + 10 * BETA (1.27, 2.07) | 25 |
| 15 | Back marking | Helper | 1 | 3 + 4.65 * BETA (1.55, 2.76) | 25 |
| 16 | Back patch pressing | Iron press | 1 | TRIA (3, 8.29, 9.73) | 25 |
| 17 | Back patch attach | S/NL^d^ | 2 | 10 + 11 * BETA (0.737, 0.96) | 25 |
| 18 | Hip pocket cutting | AWM^f^ | 1 | TRIA (3.17, 3.99, 7) | 25 |
| 19 | Hip pocket overlocks | O/L^c^ | 1 | 5 + 3.83 * BETA (2.14, 3.14) | 25 |
| 20 | Hip flap folding | Helper | 1 | NORM (4.77, 0.65) | 25 |
| 21 | Button Hole on hip flap | BH^b^ | 1 | 3.55 + GAMM (0.194, 5.47) | 25 |
| 22 | Hip flap runstitch | S/NL^d^ | 1 | 3 + LOGN (2.72, 1.83) | 25 |
| 23 | Hip flap turning | TM^g^ | 1 | NORM (3.25, 0.551) | 25 |
| 24 | Hip flap topstitches | S/NL^d^ | 1 | 3 + 5 * BETA (1.7, 1.88) | 25 |
| 25 | Hip flap attach | S/NL^d^ | 2 | 5.45 + LOGN (1.44, 0.936)  19 + 10 * BETA (1.46, 1.46) | 25 |
| 26 | Hip pocket finish |  |  |  |  |
| 27 | Back prep bundling | Helper | 1 | 3 + 2 * BETA (0.889, 0.968) | 25 |
| 28 | Front and back bundling | Helper | 1 | 2 + 6.86 * BETA (1.18, 2.11) | 25 |
| 29 | Side seam overlock | O/L^c^ | 2 | NORM (1.21, 0.115) | Not bundled |
| 30 | Side seam topstitch | F/A^h^ | 2 | TRIA (0.52, 0.747, 0.94) | Not bundled |
| 31 | Knee pocket point marking | Helper | 1 | 0.32 + 0.57 * BETA (0.889, 1.18) | Not bundled |
| 32 | Knee pocket topstitch | S/NL^d^ | 2 | 11 + ERLA (1.89, 2) | 25 |
| 33 | Knee pocket tacking | S/NL^d^ | 1 | 4 + 3 * BETA (1.33, 1.75) | 25 |
| 34 | Knee pocket Overlock | O/L^c^ | 1 | 2 + 4 * BETA (0.831, 2.05) | 25 |
| 35 | Knee pocket hemming | S/NL^d^ | 1 | 2 + 4 * BETA (1.41, 1.13) | 25 |
| 36 | Knee pocket ironing | Iron press | 2 | 8 + 5.78 * BETA (0.957, 1.06) | 25 |
| 37 | Knee pocket attach | S/NL^d^ | 2 | 0.88 + 0.92 * BETA (1.77, 1.96) | Not bundled |
| 38 | Knee flap folding | Helper | 1 | 3.63 + 3.13 * BETA (3.89, 2.38) | 25 |
| 39 | Button hole on knee flap | BH^b^ | 1 | 4.27 + WEIB (1.21, 1.99) | 25 |
| 40 | Knee flap runstitch | S/NL^d^ | 1 | TRIA (2.37, 3.81, 6.88) | 25 |
| 41 | Knee flap turning | TM^g^ | 1 | NORM (4.02, 1.01) | 25 |
| 42 | Knee flap topstitch | S/NL^d^ | 1 | 4 + 5.78 * BETA (0.903, 2.11) | 25 |
| 43 | Knee flap attach | D/NL^i^ | 2 | TRIA (0.67, 1.04, 1.7) | Not bundled |
| 44 | Bar tacking | BT^j^ | 2 | NORM (1.25, 0.266) | Not bundled |
| 45 | Back rise overlocks | O/L^c^ | 1 | 0.26 + LOGN (0.185, 0.0881) | Not bundled |
| 46 | Back rise Topstitch | D/NL^i^ | 1 | NORM (0.439, 0.0494) | Not bundled |
| 47 | Big loop matching | Helper | 1 | NORM (0.0663, 0.018) | Not bundled |
| 48 | Big loop runstitch | S/NL^d^ | 3 | 0.12 + 0.3 * BETA (2.89, 5.28) | Not bundled |
| 49 | Big loop turning | TM^g^ | 2 | 0.07 + GAMM (0.0143, 7.47) | Not bundled |
| 50 | Big loop runstitch | S/NL^d^ | 2 | 0.09 + 0.19 * BETA (1.78, 2) | Not bundled |
| 51 | Big loop button hole | BH^b^ | 1 | TRIA (0.04, 0.055, 0.11) | Not bundled |
| 52 | Small loop runstitch | LM^k^ | 1 | TRIA (0.11, 0.134, 0.18) | Not bundled |
| 53 | Small loop, big loop and waist band attach | S/NL^d^ | 3 | 1.58 + ERLA (0.068, 7) | Not bundled |
| 54 | Waist band topstitch | S/NL^d^ | 2 | TRIA (0.73, 1.34, 1.5) | Not bundled |
| 55 | Waist band closing with size labels and company tags | S/NL^d^ | 2 | 0.77 + GAMM (0.0607, 3.58) | Not bundled |
| 56 | Inseam overlock | O/L^c^ | 2 | 0.49 + WEIB (0.483, 6.16) | Not bundled |
| 57 | Trouser turning | Helper | 1 | 0.2 + LOGN (0.218, 0.112) | Not bundled |
| 58 | Inseam topstitch | F/A^h^ | 2 | 0.32 + 0.56 * BETA (1.98, 1.61) | Not Bundled |
| 59 | Button hole on hip band | BH^b^ | 1 | TRIA (0.31, 0.344, 0.47) | Not bundled |
| 60 | Button hole on the bottom leg | BH^b^ | 1 | 0.32 + 0.2 * BETA (2.7, 3.33) | Not bundled |
| 61 | Bottom rope attach | Helper | 1 | 0.5 + LOGN (0.251, 0.168) | Not bundled |
| 62 | Bottom hemming | S/NL^d^ | 2 | 0.71 + 0.73 * BETA (2.04, 2.6) | Not bundled |
| 63 | Small loop tacking | S/NL^d^ | 2 | TRIA (0.82, 1.17, 1.37) | Not bundled |
| 64 | Final bartacking | BT^j^ | 2 | TRIA (0.74, 0.851, 1.05) | Not bundled |
| 65 | Adjustable rope cutting | Helper | 1 | TRIA (0.1, 0.145, 0.19) | Not bundled |
| 66 | Adjustable hemming | S/NL^d^ | 1 | TRIA (0.1, 0.136, 0.2) | Not bundled |
| 67 | 1^st^ adjustable rope attach | S/NL^d^ | 1 | NORM (0.75, 0.0479) | Not bundled |
| 68 | 2^nd^ adjustable rope attach | S/NL^d^ | 1 | 0.53 + 0.32 * BETA (3.19, 2.1) | Not bundled |
| 69 | Button point marking | Helper | 1 | 0.55 + GAMM (0.0328, 6.16) | Not bundled |
| 70 | Trimming | Helper | 7 | NORM (4.84, 0.345) | Not bundled |
| 71 | Quality checking | Quality personnel | 2 | 0.82 + LOGN (0.332, 0.154) | Not bundled |
| 72 | Rework | S/NL^d^ | 1 | TRIA (2, 3.5, 4.7) | Not bundled |
