# Supplementary material for: Simulation metamodeling approach to complex design of garment assembly lines
Source: PLoS One. 2020 Sep 21;15(9):e0239410. doi: 10.1371/journal.pone.0239410 (PMC7505436; doi:10.1371/journal.pone.0239410)
Supplement: S3 Table — (DOCX) [file pone.0239410.s005.docx]

| **OPN^a^** | **Operations description** | **Resource** | **Quantity** | **Processing time distribution per resource** | **Bundle size** |
| --- | --- | --- | --- | --- | --- |
| 1 | Left flybox pressing | Iron press | 1 | TRIA (5, 8, 9.44) | 40 |
| 2 | Buttonhole on Left flybox | BH^b^ | 1 | 10 + WEIB (3.87, 2.34) | 40 |
| 3 | Left front rise overlock | O/L^c^ | 1 | NORM (10.4, 2.09) | 40 |
| 4 | Right front rise overlocks |  |  | NORM (5.58, 0.805) | 40 |
| 5 | Knee patch attach | S/NL^d^ | 3 | 32 + 34 * BETA (0.868, 1.4) | 40 |
| 6 | Side pocket flatlock | F/L^e^ | 2 | 6.24 + 6.72 * BETA (2.09, 2.94) | 40 |
| 7 | Side pocket overlocks | O/L^c^ | 1 | 3 + LOGN (2.03, 1.45) | 40 |
| 8 | Right flybox overlock |  |  | 2.55 + LOGN (1.16, 0.664) | 40 |
| 9 | Side pocket attach | S/NL^d^ | 2 | 12 + 17 * BETA (1.43, 1.61) | 40 |
| 10 | Side pocket topstitch | S/NL^d^ | 2 | 17 + ERLA (2.61, 2) | 40 |
| 11 | Right flybox attach | S/NL^d^ | 2 | TRIA (20, 32.9, 40) | 40 |
| 12 | Left fly box tacking | S/NL^d^ | 2 | NORM (19.2, 2.39) | 40 |
| 13 | Fly attach | S/NL^d^ | 2 | 20 + WEIB (5.97, 1.9) | 40 |
| 14 | Front prep bundling | Helper | 1 | 8 + 15 * BETA (1.11, 1.67) | 40 |
| 15 | Back marking | Helper | 1 | 5 + 7 * BETA (1.2, 2.26) | 40 |
| 16 | Back patch pressing | Iron press | 1 | 5 + 10 * BETA (1.31, 1.07) | 40 |
| 17 | Back patch attach | S/NL^d^ | 2 | 16 + 18 * BETA (0.766, 1.04) | 40 |
| 18 | Hip pocket cutting | AWM^f^ | 1 | 5.07 + ERLA (0.937, 3) | 40 |
| 19 | Hip pocket overlocks | O/L^c^ | 1 | 8 + 6 * BETA (1.97, 2.83) | 40 |
| 20 | Hip flap folding | Helper | 1 | NORM (7.63, 1.04) | 40 |
| 21 | Button Hole on hip flap | BH^b^ | 1 | NORM (7.38, 0.687) | 40 |
| 22 | Hip flap runstitch | S/NL^d^ | 1 | 5 + LOGN (4.18, 3.1) | 40 |
| 23 | Hip flap turning | TM^g^ | 1 | NORM (5.19, 0.881) | 40 |
| 24 | Hip flap topstitches | S/NL^d^ | 1 | 4.04 + 8.96 * BETA (2.41, 2.34) | 40 |
| 25 | Hip flap attach | S/NL^d^ | 2 | 9 + LOGN (2.07, 1.77)  NORM (41.9, 5.51) | 40 |
| 26 | Hip pocket finish |  |  |  |  |
| 27 | Back prep bundling | Helper | 1 | TRIA (4.47, 6.24, 8) | 40 |
| 28 | Front and back bundling | Helper | 1 | 3 + 11 * BETA (1.28, 2.11) | 40 |
| 29 | Side seam overlock | O/L^c^ | 2 | NORM (1.21, 0.115) | Not bundled |
| 30 | Side seam topstitch | F/A^h^ | 2 | TRIA (0.52, 0.747, 0.94) | Not bundled |
| 31 | Knee pocket point marking | Helper | 1 | 0.32 + 0.57 * BETA (0.889, 1.18) | Not bundled |
| 32 | Knee pocket topstitch | S/NL^d^ | 2 | 18 + 16 * BETA (1.07, 1.96) | 40 |
| 33 | Knee pocket tacking | S/NL^d^ | 1 | TRIA (6, 7.22, 11.7) | 40 |
| 34 | Knee pocket overlock | O/L^c^ | 1 | 3 + ERLA (1.02, 2) | 40 |
| 35 | Knee pocket hemming | S/NL^d^ | 1 | TRIA (3, 7.5, 10) | 40 |
| 36 | Knee pocket ironing | Iron press | 2 | 12 + 10 * BETA (1.31, 1.22) | 40 |
| 37 | Knee pocket attach | S/NL^d^ | 2 | 0.88 + 0.92 * BETA (1.77, 1.96) | Not bundled |
| 38 | Knee flap folding | Helper | 1 | 6 + 4.82 * BETA (3.53, 2.31) | 40 |
| 39 | Button hole on knee flap | BH^b^ | 1 | 7 + WEIB (1.73, 1.74) | 40 |
| 40 | Knee flap runstitch | S/NL^d^ | 1 | TRIA (4, 5.5, 11) | 40 |
| 41 | Knee flap turning | TM^g^ | 1 | 3 + WEIB (3.87, 2.24) | 40 |
| 42 | Knee flap topstitch | S/NL^d^ | 1 | 6 + ERLA (1.58, 2) | 40 |
| 43 | Knee flap attach | D/NL^i^ | 2 | TRIA (0.67, 1.04, 1.7) | Not bundled |
| 44 | Bar tacking | BT^j^ | 2 | NORM (1.25, 0.266) | Not bundled |
| 45 | Back rise overlocks | O/L^c^ | 1 | 0.26 + LOGN (0.185, 0.0881) | Not bundled |
| 46 | Back rise Topstitch | D/NL^i^ | 1 | NORM (0.439, 0.0494) | Not bundled |
| 47 | Big loop matching | Helper | 1 | NORM (0.0663, 0.018) | Not bundled |
| 48 | Big loop runstitch | S/NL^d^ | 3 | 0.12 + 0.3 * BETA (2.89, 5.28) | Not bundled |
| 49 | Big loop turning | TM^g^ | 2 | 0.07 + GAMM (0.0143, 7.47) | Not bundled |
| 50 | Big loop runstitch | S/NL^d^ | 2 | 0.09 + 0.19 * BETA (1.78, 2) | Not bundled |
| 51 | Big loop button hole | BH^b^ | 1 | TRIA (0.04, 0.055, 0.11) | Not bundled |
| 52 | Small loop runstitch | LM^k^ | 1 | TRIA (0.11, 0.134, 0.18) | Not bundled |
| 53 | Small loop, big loop and waistband attach | S/NL^d^ | 3 | 1.58 + ERLA (0.068, 7) | Not bundled |
| 54 | Waistband topstitch | S/NL^d^ | 2 | TRIA (0.73, 1.34, 1.5) | Not bundled |
| 55 | Waist band closing with size and label tags | S/NL^d^ | 2 | 0.77 + GAMM (0.0607, 3.58) | Not bundled |
| 56 | Inseam overlock | O/L^c^ | 2 | 0.49 + WEIB (0.483, 6.16) | Not bundled |
| 57 | Trouser turning | Helper | 1 | 0.2 + LOGN (0.218, 0.112) | Not bundled |
| 58 | Inseam topstitch | F/A^h^ | 2 | 0.32 + 0.56 * BETA (1.98, 1.61) | Not Bundled |
| 59 | Button hole on hip band | BH^b^ | 1 | TRIA (0.31, 0.344, 0.47) | Not bundled |
| 60 | Button hole on the bottom leg | BH^b^ | 1 | 0.32 + 0.2 * BETA (2.7, 3.33) | Not bundled |
| 61 | Bottom rope attach | Helper | 1 | 0.5 + LOGN (0.251, 0.168) | Not bundled |
| 62 | Bottom hemming | S/NL^d^ | 2 | 0.71 + 0.73 * BETA (2.04, 2.6) | Not bundled |
| 63 | Small loop tacking | S/NL^d^ | 2 | TRIA (0.82, 1.17, 1.37) | Not bundled |
| 64 | Final bartacking | BT^j^ | 2 | TRIA (0.74, 0.851, 1.05) | Not bundled |
| 65 | Adjustable rope cutting | Helper | 1 | TRIA (0.1, 0.145, 0.19) | Not bundled |
| 66 | Adjustable hemming | S/NL^d^ | 1 | TRIA (0.1, 0.136, 0.2) | Not bundled |
| 67 | 1^st^ adjustable rope attach | S/NL^d^ | 1 | NORM (0.75, 0.0479) | Not bundled |
| 68 | 2^nd^ adjustable rope attach | S/NL^d^ | 1 | 0.53 + 0.32 * BETA (3.19, 2.1) | Not bundled |
| 69 | Button point marking | Helper | 1 | 0.55 + GAMM (0.0328, 6.16) | Not bundled |
| 70 | Trimming | Helper | 7 | NORM (4.84, 0.345) | Not bundled |
| 71 | Quality checking | Quality personnel | 2 | 0.82 + LOGN (0.332, 0.154) | Not bundled |
| 72 | Rework | S/NL^d^ | 1 | TRIA (2, 3.5, 4.7) | Not bundled |
