# Supplementary material for: Simulation metamodeling approach to complex design of garment assembly lines
Source: PLoS One. 2020 Sep 21;15(9):e0239410. doi: 10.1371/journal.pone.0239410 (PMC7505436; doi:10.1371/journal.pone.0239410)
Supplement: S4 Table — (DOCX) [file pone.0239410.s006.docx]

| **Real-world throughput data** | | |
| --- | --- | --- |
| s/n | Date | Throughput (pieces per day) |
| 1 | 26/03/2019 | 239 |
| 2 | 27/03/2019 | 570 |
| 3 | 28/03/2019 | 430 |
| 4 | 29/03/2019 | 580 |
| 5 | 30/03/2019 | 600 |
| 6 | 01/04/2019 | 570 |
| 7 | 02/04/2019 | 464 |
| 8 | 03/04/2019 | 440 |
| 9 | 04/04/2019 | 306 |
| 10 | 05/04/2019 | 544 |
| 11 | 06/04/2019 | 347 |
| 12 | 09/04/2019 | 350 |
| 13 | 10/04/2019 | 600 |
| 14 | 11/04/2019 | 650 |
| 15 | 12/04/2019 | 580 |
| 16 | 13/04/2019 | 600 |
| 17 | 14/04/2019 | 224 |
| 18 | 15/04/2019 | 468 |
| 19 | 18/04/2019 | 512 |
| 20 | 19/04/2019 | 500 |
| 21 | 20/04/2019 | 512 |
| 22 | 23/04/2019 | 552 |
| 23 | 24/04/2019 | 650 |
