# Supplementary material for: Simulation metamodeling approach to complex design of garment assembly lines
Source: PLoS One. 2020 Sep 21;15(9):e0239410. doi: 10.1371/journal.pone.0239410 (PMC7505436; doi:10.1371/journal.pone.0239410)
Supplement: S5 Table — (DOCX) [file pone.0239410.s007.docx]

| Cutting section trouser parts feeding sheet | | | | | |
| --- | --- | --- | --- | --- | --- |
| Date | S/N | Bundle number | Trouser size | Quantity | Total |
| 29/03/2019 | 1 | 233 |  | 25 |  |
|  |  | 234 |  | 25 |  |
|  |  | 235 |  | 25 |  |
|  |  | 236 |  | 25 |  |
|  |  | 237 | L1 | 25 |  |
|  |  | 238 |  | 25 |  |
|  |  | 239 |  | 25 |  |
|  |  | 240 |  | 25 |  |
|  |  |  |  |  | 200 |
|  | 2 | 241 |  | 25 |  |
|  |  | 242 |  | 25 |  |
|  |  | 243 |  | 25 |  |
|  |  | 244 | L2 | 25 |  |
|  |  | 245 |  | 25 |  |
|  |  | 246 |  | 25 |  |
|  |  | 247 |  | 25 |  |
|  |  | 248 |  | 25 |  |
|  |  |  |  |  | 200 |
|  | 3 | 249 |  | 25 |  |
|  |  | 250 |  | 25 |  |
|  |  | 251 |  | 25 |  |
|  |  | 252 |  | 25 |  |
|  |  | 253 | XL1 | 25 |  |
|  |  | 254 |  | 25 |  |
|  |  | 255 |  | 25 |  |
|  |  | 256 |  | 25 |  |
|  |  |  |  |  | 200 |
|  | 4 | 257 |  | 25 |  |
|  |  | 258 |  | 25 |  |
|  |  | 259 |  | 25 |  |
|  |  | 260 | XL2 | 25 |  |
|  |  | 261 |  | 25 |  |
|  |  | 262 |  | 25 |  |
|  |  | 263 |  | 25 |  |
|  |  | 264 |  | 25 |  |
|  |  |  |  |  | 200  = **800** |

| **Cutting section trouser parts feeding sheet** | | | | | |
| --- | --- | --- | --- | --- | --- |
| Date | S/N | Bundle size | Trouser size | Quantity | Total |
| 10.04.2019 | 16 | 281 |  | 25 |  |
|  |  | 282 |  | 25 |  |
|  |  | 283 |  | 25 |  |
|  |  | 284 | S1 | 25 |  |
|  |  | 285 |  | 25 |  |
|  |  | 286 |  | 25 |  |
|  |  | 287 |  | 27 |  |
|  |  | 288 |  | 28 |  |
|  |  |  |  |  | 205 |
|  | 17. | 289 |  | 25 |  |
|  |  | 290 |  | 25 |  |
|  |  | 291 | S2 | 25 |  |
|  |  | 292 |  | 25 |  |
|  |  | 293 |  | 25 |  |
|  |  | 294 |  | 25 |  |
|  |  | 295 |  | 27 |  |
|  |  | 296 |  | 28 |  |
|  |  |  |  |  | 205 |
|  | 18. | 297 |  | 25 |  |
|  |  | 298 |  | 25 |  |
|  |  | 299 |  | 25 |  |
|  |  | 300 | S | 25 |  |
|  |  | 301 |  | 25 |  |
|  |  | 302 |  | 25 |  |
|  |  | 303 |  | 27 |  |
|  |  | 304 |  | 28 |  |
|  |  |  |  |  | 205 |
|  | 19. | 305 |  | 25 |  |
|  |  | 306 |  | 25 |  |
|  |  | 307 |  | 25 |  |
|  |  | 308 | 2XL | 25 |  |
|  |  | 309 |  | 25 |  |
|  |  | 310 |  | 25 |  |
|  |  | 311 |  | 27 |  |
|  |  | 312 |  | 28 |  |
|  |  |  |  |  | 205  = **820** |
