# Supplementary material for: Simulation metamodeling approach to complex design of garment assembly lines
Source: PLoS One. 2020 Sep 21;15(9):e0239410. doi: 10.1371/journal.pone.0239410 (PMC7505436; doi:10.1371/journal.pone.0239410)
Supplement: Attachment — Submitted filename: PONE Rebuttal Letter final.docx [file pone.0239410.s009.docx]

MOI UNIVERSITY

**SCHOOL OF ENGINEERING**

**DEPARTMENT OF MANUFACTURING, INDUSTRIAL AND TEXTILE ENGINEERING**

Tel. +254706536659

Website.www.mu.ac.ke

ww[www.mu.ac.ke](http://www.mu.ac.ke)

P.O. Box 3900,

Eldoret, Kenya

8^th^ July, 2020

Dear **Editor in Chief,**

***PLOS ONE***

We are grateful to the Editorial team and the Reviewers for the valuable time, useful contributions and generous comments accorded to our manuscript PONE-D-20-13481: “*Simulation metamodeling approach to complex design of garment assembly lines*”. We have revised the manuscript in response to the extensive and insightful review comments. The introduction, methodology, discussion and conclusion sections in the manuscript have been revised. English language throughout the manuscript have been checked. We have responded to the review comments point-counter-point and as advised, we have attempted to succinctly explain the changes made in reaction to all the review comments. Changes in the revised manuscript are highlighted in yellow. In the rebuttal provided on the next page, concerns raised are shown as normal text while our responses appear as colored text (blue).

We look forward to hearing from you regarding our resubmission. We shall be glad to respond to any further questions and comments that the review team may raise to improve the quality of our manuscript.

Thank you for your time and consideration in advance,

**Ocident Bongomin** (On behalf of all authors)

**JOURNAL REQUIREMENTS:**

When submitting your revision, we need you to address these additional requirements.

1. Please ensure that your manuscript meets PLOS ONE's style requirements, including those for file naming. The PLOS ONE style templates can be found at  <https://journals.plos.org/plosone/s/file?id=wjVg/PLOSOne_formatting_sample_main_body.pdf> and <https://journals.plos.org/plosone/s/file?id=ba62/PLOSOne_formatting_sample_title_authors_affiliations.pdf>

Thank you for this recommendation. We have revised the manuscript as per the suggested journal style requirements.

We suggest you thoroughly copyedit your manuscript for language usage, spelling, and grammar. If you do not know anyone who can help you do this, you may wish to consider employing a professional scientific editing service. Whilst you may use any professional scientific editing service of your choice, PLOS has partnered with both American Journal Experts (AJE) and Editage to provide discounted services to PLOS authors. Both organizations have experience helping authors meet PLOS guidelines and can provide language editing, translation, manuscript formatting, and figure formatting to ensure your manuscript meets our submission guidelines. To take advantage of our partnership with AJE, visit the AJE website (<http://learn.aje.com/plos/>) for a 15% discount off AJE services. To take advantage of our partnership with Editage, visit the Editage website ([www.editage.com](http://www.editage.com/)) and enter referral code PLOSEDIT for a 15% discount off Editage services.  If the PLOS editorial team finds any language issues in text that either AJE or Editage has edited, the service provider will re-edit the text for free.

The manuscript has been checked for grammatical fixes and English language by a proficient colleague.

Upon resubmission, please provide the following:

- The name of the colleague or the details of the professional service that edited your manuscript

The manuscript was proofread by Timothy Omara, an experienced researcher with the Department of Chemistry and Biochemistry, Moi University, Kenya. We have added this to the acknowledgement section of the revised manuscript.

- A copy of your manuscript showing your changes by either highlighting them or using track changes (uploaded as a *supporting information* file)

We have uploaded a copy of the manuscript with highlighted changes as a supporting information file.

- A clean copy of the edited manuscript (uploaded as the new *manuscript* file)

This has been uploaded as suggested.

2. We note that in the Data Availability statement you have provided a link to the dataset used in this study. We ask that you additionally provide this link within the Methods section for the convenience of the reader.

Thank you for this suggestion. We have included the link to the dataset in the methodology section (page 9, Line 191 and page 14, Line 277).

**ADDITIONAL EDITOR COMMENTS (IF PROVIDED):**

Based on the reviewers' comments, I suggest the authors to do a major revision for this paper and submit a detailed response, so we can further consider if it is suitable to accept this paper.
[Note: HTML markup is below. Please do not edit.]

We have done extensive revision of the manuscript closely following the reviewers’ comments. Thank you.

**REVIEWERS' COMMENTS:**

# Reviewer's Responses to Questions

1. Is the manuscript technically sound, and do the data support the conclusions?
The manuscript must describe a technically sound piece of scientific research with data that supports the conclusions. Experiments must have been conducted rigorously, with appropriate controls, replication, and sample sizes. The conclusions must be drawn appropriately based on the data presented.

Reviewer #1: Yes

Reviewer #2: Partly

2. Has the statistical analysis been performed appropriately and rigorously?

Reviewer #1: Yes

Reviewer #2: Yes

3. Have the authors made all data underlying the findings in their manuscript fully available?
The [PLOS Data policy](http://www.plosone.org/static/policies.action#sharing) requires authors to make all data underlying the findings described in their manuscript fully available without restriction, with rare exception (please refer to the Data Availability Statement in the manuscript PDF file). The data should be provided as part of the manuscript or its supporting information, or deposited to a public repository. For example, in addition to summary statistics, the data points behind means, medians and variance measures should be available. If there are restrictions on publicly sharing data—e.g. participant privacy or use of data from a third party—those must be specified.

Reviewer #1: Yes

Reviewer #2: No

4. Is the manuscript presented in an intelligible fashion and written in standard English?
PLOS ONE does not copyedit accepted manuscripts, so the language in submitted articles must be clear, correct, and unambiguous. Any typographical or grammatical errors should be corrected at revision, so please note any specific errors here.

Reviewer #1: Yes

Reviewer #2: Yes

5. **Review Comments to the Author**

Please use the space provided to explain your answers to the questions above. You may also include additional comments for the author, including concerns about dual publication, research ethics, or publication ethics. (Please upload your review as an attachment if it exceeds 20,000 characters)

**Reviewer #1:**

(1) The paper is well written and includes interesting findings for the engineers. I do not have any specific comment, however, I recommend improving the figures quality, and writing level. Also please clearly specify the contribution at the end of introduction section. Try to condense the theoretical parts and use the flowcharts as much as possible for illustrative purposes.

Thank you for the insightful comments. We have improved the resolution of the figures. The manuscript have been proofread by a proficient colleague. The contribution of our study has also been added to the introduction section as advised (page 6, Lines 124-126)

We have included a flowchart (Fig 1; page 7) in the methodology section to further describe the approach used in the study.

(2) Literature review must include application of Latin hypercube design and orthogonal array for design of experiment. Further, if profound metamodeling technique involving machine learning approach is used in this domain must be cited in introduction of paper.

This is quite correct. We have included the application of Latin hypercube design and orthogonal array on page 5, Lines107-109 (introduction section). We have also cited the use of machine learning technique (e.g. Artificial neural network) for metamodeling on page 5, Lines 94-96.

**Reviewer #2:**

I believe this paper presents an interesting piece of work. I am not particularly very familiar with the garment manufacturing and research literature, as I am with the simulation metamodeling framework in general. The authors mention that simulation is a commonly applied tool within the garment and assembly line industries. However, the same does not hold for simulation metamodels. For this reason, i.e., for using a technique that is not (yet) often applied within a particular field, I think that this work already has some merit.

Thank you for this commendation.

On the other hand, there is much room for improvement.

First of all, the pictures/figures used. I found two problems: their bad quality or low resolution. For instance, I can barely read the contents of Figure 2. Secondly: I do not find it very comfortable for the figures to be at the end of the paper. The constant scroll up/down between the figures and the text where they are referenced is very distracting and demotivating. The authors should try to solve these two problems.

Firstly, we apologize for this inconvenience. Higher resolution figures have been supplied. For the positioning of the figures, these were done in accordance with PLoS Ones’ manuscript preparation guidelines. For this reason, we could not reposition these figures. Thank you.

The paper is generally well written, but the writing style and some typos should be reviewed here and there. Some examples (with correction/suggestion): "However, most of THE simulation studies have considered ill-defined experimental designS..."; "Simulation has been majorly applied for THE analysis of complex systems to give extensive insightS..." (?)

This is quite correct. The manuscript has been extensively revised to eliminate typos, grammatical and spelling errors.

Here "Metamodels, also well-known as meta-models, surrogate models, or emulators are used to approximate the input-output behavior of simulation models.", I would remove the reference to "meta-models" since metamodels = meta-models (it is just a different way of writing the same thing, although I think "metamodel" is more common). I would add "response surfaces" too.

Thank you for this expert suggestion. We removed meta-models and added response surfaces as suggested.

In line 62, the authors mention that metamodeling aims to reduce the computation costs of simulation models during the optimization process. This is only partially true. It is true if the metamodeling strategy is being employed within the simulation-optimization approaches, but it is not valid if there is no optimization process involved. In general, simulation metamodels aim to bypass the simulation model itself yes!

Very well, we have removed this statement and included the general advantages of metamodeling in optimization (page 4 line 86 and page 5 lines 87-88)

This paper "Jack PC Kleijnen and Wim CM Van Beers. Application-driven sequential designs for simulation experiments: Kriging metamodeling. Journal of the Operational Research Society, 55(8):876–883, 2004.", elegantly identifies the four primary objectives of simulation metamodels. Speaking of other papers, I think that the literature review around simulation models could be expanded a bit, as it is the central theme of this work.

We have reviewed more papers including the suggested paper and others on simulation models. We have expanded this part of the manuscript (please check page 3, Lines 53-64 and page 5, Lines 91-96). We hope this is agreeable.

I could understand that the Fractional Factorial design was used, but it is not very clear to me what type of regression model was used as metamodel. Was it the Multiple Linear Regression model? I could infer this from Equations 1 and 2.

You are very right. It is a multiple linear regression model with linear terms and two-way interactions that we used. Thank you

These are my comments for now.

6. PLOS authors have the option to publish the peer review history of their article ([what does this mean?](https://journals.plos.org/plosone/s/editorial-and-peer-review-process#loc-peer-review-history)). If published, this will include your full peer review and any attached files.
If you choose “no”, your identity will remain anonymous but your review may still be made public.
**Do you want your identity to be public for this peer review?** For information about this choice, including consent withdrawal, please see our [Privacy Policy](https://www.plos.org/privacy-policy).

Reviewer #1: **Yes:** Ramin Ghiasi

Reviewer #2: No
[NOTE: If reviewer comments were submitted as an attachment file, they will be attached to this email and accessible via the submission site. Please log into your account, locate the manuscript record, and check for the action link "View Attachments". If this link does not appear, there are no attachment files.]

While revising your submission, please upload your figure files to the Preflight Analysis and Conversion Engine (PACE) digital diagnostic tool, <https://pacev2.apexcovantage.com/>. PACE helps ensure that figures meet PLOS requirements. To use PACE, you must first register as a user. Registration is free. Then, login and navigate to the UPLOAD tab, where you will find detailed instructions on how to use the tool. If you encounter any issues or have any questions when using PACE, please email PLOS at [figures@plos.org](mailto:figures@plos.org). Please note that Supporting Information files do not need this step.
