# Supplementary material for: Simulation metamodeling approach to complex design of garment assembly lines
Source: PLoS One. 2020 Sep 21;15(9):e0239410. doi: 10.1371/journal.pone.0239410 (PMC7505436; doi:10.1371/journal.pone.0239410)
Supplement: Attachment — Submitted filename: PONE Rebuttal Letter final 2.docx [file pone.0239410.s010.docx]

MOI UNIVERSITY

**SCHOOL OF ENGINEERING**

**DEPARTMENT OF MANUFACTURING, INDUSTRIAL AND TEXTILE ENGINEERING**

Tel. +254706536659

Website.www.mu.ac.ke

ww[www.mu.ac.ke](http://www.mu.ac.ke)

P.O. Box 3900,

Eldoret, Kenya

27^th^ August, 2020

Dear **Editor in Chief,**

***PLOS ONE***

We are grateful to the Editorial team and the Reviewers for the valuable time, useful contributions and generous comments accorded to our manuscript PONE-D-20-13481R1: “*Simulation metamodeling approach to complex design of garment assembly lines*”. We have revised the manuscript in response to the review comments. English language throughout the manuscript have been checked. We have responded to the review comments point-counter-point and as advised, we have attempted to succinctly explain the changes made in reaction to all the review comments. Changes in the revised manuscript are highlighted in yellow. In the rebuttal provided on the next page, concerns raised are shown as normal text while our responses appear as colored text (blue).

We look forward to hearing from you regarding our resubmission. We shall be glad to respond to any further questions and comments that the review team may raise to improve the quality of our manuscript.

Thank you for your time and consideration in advance,

**Ocident Bongomin** (On behalf of all authors)

**ADDITIONAL EDITOR COMMENTS (IF PROVIDED):**

Based on the review comments, I would like to accept this paper after you make a minor revision according to the reviewer's comments.

We have revised the manuscript closely to the reviewer’s comments. Thank you

[Note: HTML markup is below. Please do not edit.]

**REVIEWERS' COMMENTS:**

Reviewer's Responses to Questions

**Comments to the Author**

1. If the authors have adequately addressed your comments raised in a previous round of review and you feel that this manuscript is now acceptable for publication, you may indicate that here to bypass the “Comments to the Author” section, enter your conflict of interest statement in the “Confidential to Editor” section, and submit your "Accept" recommendation.

Reviewer #1: All comments have been addressed

Reviewer #2: All comments have been addressed

2. Is the manuscript technically sound, and do the data support the conclusions?
The manuscript must describe a technically sound piece of scientific research with data that supports the conclusions. Experiments must have been conducted rigorously, with appropriate controls, replication, and sample sizes. The conclusions must be drawn appropriately based on the data presented.

Reviewer #1: Yes

Reviewer #2: Partly

3. Has the statistical analysis been performed appropriately and rigorously?

Reviewer #1: Yes

Reviewer #2: Yes

4. Have the authors made all data underlying the findings in their manuscript fully available?
The [PLOS Data policy](http://www.plosone.org/static/policies.action#sharing) requires authors to make all data underlying the findings described in their manuscript fully available without restriction, with rare exception (please refer to the Data Availability Statement in the manuscript PDF file). The data should be provided as part of the manuscript or its supporting information, or deposited to a public repository. For example, in addition to summary statistics, the data points behind means, medians and variance measures should be available. If there are restrictions on publicly sharing data—e.g. participant privacy or use of data from a third party—those must be specified.

Reviewer #1: Yes

Reviewer #2: No

5. Is the manuscript presented in an intelligible fashion and written in standard English?
PLOS ONE does not copyedit accepted manuscripts, so the language in submitted articles must be clear, correct, and unambiguous. Any typographical or grammatical errors should be corrected at revision, so please note any specific errors here.

Reviewer #1: Yes

Reviewer #2: Yes

6. Review Comments to the Author

Please use the space provided to explain your answers to the questions above. You may also include additional comments for the author, including concerns about dual publication, research ethics, or publication ethics. (Please upload your review as an attachment if it exceeds 20,000 characters)

Reviewer #1: (No Response)

Reviewer #2: My comments have been addressed.

I recommended the paper "Jack PC Kleijnen and Wim CM Van Beers. Application-driven sequential designs for simulation experiments: Kriging metamodeling. Journal of the Operational Research Society, 55(8):876–883, 2004." stating that it explains the four main objectives of simulation metamodels, but I made a mistake. It can still be used for the literature review, of course.
Although from the same first author, the paper that I had in mind and I was recommending it was the following: "Jack PC Kleijnen and Robert G Sargent. A methodology for fitting and validating metamodels in simulation. European Journal of Operational Research, 120(1):14–29, 2000." As the title itself indicates, this paper provides a more general and comprehensive overview of the simulation metamodelling process. Apologies for this mix-up.

We have included the suggested reference in the revised manuscript. Please check page 5, line 98-100. Thank you

7. PLOS authors have the option to publish the peer review history of their article ([what does this mean?](https://journals.plos.org/plosone/s/editorial-and-peer-review-process#loc-peer-review-history)). If published, this will include your full peer review and any attached files.
If you choose “no”, your identity will remain anonymous but your review may still be made public.
**Do you want your identity to be public for this peer review?** For information about this choice, including consent withdrawal, please see our [Privacy Policy](https://www.plos.org/privacy-policy).

Reviewer #1: No

Reviewer #2: No

[NOTE: If reviewer comments were submitted as an attachment file, they will be attached to this email and accessible via the submission site. Please log into your account, locate the manuscript record, and check for the action link "View Attachments". If this link does not appear, there are no attachment files.]

While revising your submission, please upload your figure files to the Preflight Analysis and Conversion Engine (PACE) digital diagnostic tool, <https://pacev2.apexcovantage.com/>. PACE helps ensure that figures meet PLOS requirements. To use PACE, you must first register as a user. Registration is free. Then, login and navigate to the UPLOAD tab, where you will find detailed instructions on how to use the tool. If you encounter any issues or have any questions when using PACE, please email PLOS at [figures@plos.org](mailto:figures@plos.org). Please note that Supporting Information files do not need this step.
